# Comparison of chemical composition in the buds of *Aralia elata* from different geographical origins of China

**DOI:** 10.1098/rsos.180676

**Published:** 2018-08-15

**Authors:** Mingming Qi, Xiaoyu Hua, Xiaoyuan Peng, Xiufeng Yan, Jixiang Lin

**Affiliations:** 1 Alkali Soil Natural Environmental Science Center, Northeast Forestry University/Key Laboratory of Saline-alkali Vegetation Ecology Restoration, Ministry of Education, Harbin 150040, People's Republic of China; 2 Department of Plant Pathology, North Carolina State University, Raleigh, NC 27695–7716, USA

**Keywords:** *Aralia elata*, phenolic compounds, mineral elements, geographical origin

## Abstract

*Aralia elata* buds contain many nutrients and have a pleasant taste with a unique flavour. Previous studies mainly focused on triterpene saponins in the root bark of this species, but little information existed concerning other chemical components, especially in the buds. To better understand the nutritional value of *A. elata*, we compared total flavonoids, total saponins, phenolic compounds and mineral element contents in the buds of *A. elata* collected from eight different geographical regions (S1: Benxi; S2: Linjiang; S3: Pingwu; S4: Enshi; S5: Changbaishan; S6: Shangzhi; S7: Xiaoxinganling and S8: Harbin) in China. The results showed that the basic composition in the buds presented a wide variation, with ash (8.76–10.35%), crude fibre (5.38–11.07%), polysaccharides (33.85–46.79 mg g^−1^), total flavonoid content (TFC, 4.06–48.63 mg g^−1^) and total saponins (13.62–27.85 mg g^−1^). UPLC combined with the LC-MS/MS method was used for the phenolic compounds analysis, and 11 phenolic compounds were identified and quantified in the eight samples. The total phenolic content in Enshi (S4) was significantly higher than others, and quercetin was the predominant phenolic compound in this sample. We used ICP-OES to identify and quantify nine mineral elements in the buds. The Fe and Cu contents in S5 were much higher than that of others. We obtained maximum Mg, Mn, Co and Ni contents in S4, and found rich Zn content in S7. Moreover, the maximum estimated quantities of Ca and Sr were found in S8. This study indicated that the chemical composition in the buds of *A. elata* was obviously affected by geographical origin. Our results provided an essential theoretical basis of quality evaluation of *A. elata* buds in the food production field.

## 1. Introduction

*Aralia elata* (Miq.) Seem, a shrub of the Araliaceae family, widely distributed in northeastern China (Heilongjiang, Liaoning and Jilin provinces), far eastern Russia, Japan and Korea [1]. Its barks and root are widely applied in traditional folk medicine for the treatment of rheumatoid arthritis [2], diabetes mellitus [3] and hepatitis [4], etc. Also, the buds of *A. elata*, locally called ‘ci lao ya’, are always used as edible wild vegetables because of their unique flavour and good taste, while containing many nutrients including proteins, carbohydrates and vitamins [5]. For the above reasons, increasing attention is being paid to this plant.

Phenolic compounds, one of the most critical components contributing to the quality of wild vegetables, exhibit various biological activities, such as eliminating free radicals, inhibiting oxidation, microbial growth and anti-cancer activity [6,7]. For example, in *Zizania aquatic* species, the main contributors to antioxidant activity are ferulic, vanillic, ellagic, sinapic and syringic acids. Regarding free flavonoids, the main contributors to antioxidant activity are epigallocatechin, epicatechin and rutin [8]. The phenolic extracts from propolis exhibited a certain level of *in vitro* anti-mutagenic activity in lymphocytes from healthy subjects, and anti-cancer activity in breast cancer cell lines and may be considered as safe and healthy food supplements in cancer therapy [9].

Mineral elements include macroelements (N, P, K, S, Ca and Mg) and microelements (Fe, Mn, B, Zn, Cu, Mo and Cl). P is one of the most important components of DNA and RNA molecules, which is necessary in the mechanisms of expression of specific genes, and also as the component of proteins controlling enzymatic reactions [10]. Ca, as the second messenger, can transfer information to the tissues. In complex with calmodulin, Ca can activate enzymes (protein kinases) responsible for expression of specific genes [11]. Mg is localized in the centres of the porphyrin rings of chlorophyll molecules in photosynthetic systems. For microelements, Fe, Mn, Zn and Cu are cofactors of antioxidant enzymes, especially superoxide dismutases (SODs), which can prevent peroxidation of biomolecules [12].

*Aralia elata* buds have a highly sophisticated and nutritive chemical composition, but the constituents vary within a minimum and maximum range of values, primarily due to the geographical origins. The majority of previous studies on the chemical composition of *A. elata* were mainly focused on triterpene saponins in the root bark [4,13], and the pharmacological activity of this species, such as antiviral, antioxidative and anti-cancer properties [14,15]. However, there was little information regarding other chemical components, such as the phenolic compounds and minerals, especially within the buds.

The objectives in the present study were (i) to analyse the main chemical composition (such as phenolic compounds and mineral elements) in the buds of *A. elata*; and (ii) to compare the differences in the chemical composition of the buds from different geographical origins.

## 2. Material and methods

### 2.1. Plant materials

The buds of *A. elata* studied in this work were collected from its wild habitat in May–June 2015, from various areas in China, including Benxi (Liaoning province, 40°49′ N, 123°34′ E, S1), Linjiang (Jilin province, 41°40′ N, 127°15′ E, S2), Pingwu (Sichuan province, 31°59′ N, 103°50′ E, S3), Enshi (Hubei province, 29°07′ N, 108°23′ E, S4), Changbaishan (Jilin province, 41°41′ N, 127°42′ E, S5), Shangzhi (Hei longjiang province, 45°34′ N, 128°09′ E, S6), Xiaoxinganling (Hei longjiang province, 46°28′ N, 127°44′ E, S7) and Harbin (Hei longjiang province, 44°04′ N, 125°42′ E, S8). All the obtained samples were dried at 30°C, and three biological replicates were performed.

### 2.2. Chemicals

Standards of protocatechuic acid, caffeic acid, chlorogenic acid, ferulic acid, gallic acid, catechin, epicatechin 4-hydroxybenzoic acid, quercetin, salicylic acid and scopoletin were purchased from National Institutes for Food and Drug Control, 3-Hydroxycinnamic acid was purchased from ChromaDex (USA), sinapic acid and 4-Hydroxycoumarin were purchased from Stanford Chemicals (USA). Methanol, acetonitrile and Folin phenol reagent were obtained from Sigma-Aldrich (USA). The standards of Zn, Cu, Fe, Cr, Ni, Sr, Ca and Mg were purchased from National Nonferrous Metals and Electronic Materials Analysis and Testing Center. In addition, nitric acid was electronic grade, and all other reagents used in this experiment were of analytical grade.

### 2.3. Basic composition analysis

The ash content and crude fibre determinations were measured following GB/T12532-2008 and GB/T5009.10-2003.

Polysaccharides were extracted by the traditional hot water extraction. The dried buds were ground into fine powder, and extracted with 95% ethanol for 4 h to remove lipid. The degreased powders (0.5 g) were obtained with distilled water (80 ml) under reflux for 2 h. After centrifugation (6000 r.p.m., 15 min), the supernatant was concentrated. The yield was determined by the phenol–sulfuric acid colorimetric method [16,17].

The total flavonoid content (TFC) was determined using a colorimetric method with some modifications as described in [18]. After the powders (0.1 g) were dissolved in 2 ml of 70% ethanol, ultrasonic extraction was performed for 1 h and then centrifugation for 10 min. The supernatant dilute was added with 70% ethanol to a volume of 10 ml. We took 5 ml from it, and 0.3 ml of 5% NaNO_2_ was added. The tubes were then allowed to stand for 6 min; subsequently 0.3 ml of 10% Al(NO_3_)_3_ was added to the reaction mixture, which was again allowed to stand for 6 min. Finally, 4 ml of 1 M NaOH and 0.4 ml of 70% ethanol were added and mixed immediately into the mixture, which was allowed to stand for 10 min. The absorbance was measured at 510 nm against a blank prepared similarly by replacing the extract with 70% ethanol. The TFC was then calculated according to the standard curve.

Total saponin (TS) content was determined as follows: The dry bud powders (1.0 g) were treated with 20 ml of petroleum ether for 1 h to remove the lipid. The degreased powders (0.1 g) were extracted with 70% ethanol, and ultrasonic extraction for 1 h. After centrifugation for 10 min, the supernatant was diluted with 70% ethanol to 5 ml. We took 50 µl from it, and 0.2 ml vanillin-glacial acetic acid and 0.8 ml perchloric acid were added. After placing the reaction mixture in a water bath (60°C) for 15 min, 4 ml of ethyl acetate was added. The absorbance was measured at 560 nm.

### 2.4. Phenolic compounds analysis

#### 2.4.1. Extraction

Two sets of samples (0.1 g for each) were prepared. One set of samples were extracted with 2 ml of distilled water and ultrasonic extraction for 90 min, and then centrifuged (13 000 r.p.m., 20 min). The supernatant was rotated to dry at 45°C, then 200 µl of methanol and water (1 : 1) were added, and the supernatant was used for the analysis of phenolic compounds. Another set of samples was used for the study of the total phenolic content (TPC).

#### 2.4.2. UPLC analysis of phenolic compounds

A Waters ACQUITY UPLC system (Waters, Milford, MA, USA) was used for the phenolic compounds analysis. Separation was performed on the ACQUITY UPLC HSS T3 column (2.1 × 50 mm, 1.8 µm). The mobile phase consisted of water with 0.5% acetic acid (solvent A; v/v), methanol with 0.5% acetic acid (solvent B; v/v) and acetonitrile with 0.5% acetic acid (solvent C; v/v) at a flow rate of 0.40 ml min^−1^. Samples (10 µl) were injected into the UPLC system. The gradient elution conditions started with 95% A, 4% B, 1% C to 4.0 min, adjusted to 85% A, 12% B, 3% C from 4.0 min to 6.0 min, 80% A, 16% B, 4% C from 6.0 min to 10.0 min, 70% A, 25% B, 5% C from 10.0 min to 13.0 min, 20% A, 40% B, 40% C from 13.0 min to 16.0 min, 0% A, 50% B, 50% C from 16.0 min to 19.0 min and back to the initial condition from 19.0 min to 24.0 min. The identification and assignation of each compound were performed by comparing their retention time and standard, and also confirmed by an AB Sciex LC-MS/MS spectrometer equipped with an electrospray ionization source. The electrospray ionization mass spectrometry detection was performed in the negative ion mode with the following optimized parameters: capillary voltage 2.50 kV, cone voltage 75 V, desolvation gas 650 l h^−1^, cone gas 50 l h^−1^, desolvation temperature 450°C, source temperature 150°C and extractor 3.00 V.

### 2.5. Analysis of the mineral content

The determinations of the concentrations of all of the minerals were conducted with an ICP-OES instrument (PerkinElmer, Optima 8300, USA). The operating conditions of the ICP-OES equipment were as follows: 14 l min^−1^ plasma gas flow rate, 0.2 l min^−1^ auxiliary gas flow rate, 0.55 l min^−1^ nebulizer gas flow rate, 1200 WRF power, and 1.5 ml min^−1^ sample flow rate. All elements were detected in the axial mode. The analytical wavelengths (nm) were as follows: Cr (267.716), Cu (327.393), Fe (238.204), Mn (257.610), Ni (231.604), Sr (407.771) and Zn (206.200). An ICP-OES multi-element standard containing Mn (0.1–12.5 mg l^−1^), Ni (0.1–12.5 mg l^−1^), Sr (0.1–12.5 mg l^−1^), Fe (0.1–12.5 mg l^−1^), Cu (0.1–12.5 mg l^−1^) and Zn (0.1–12.5 mg l^−1^) was prepared. Cr was prepared as a series of single-element standards from 0.1 mg l^−1^ to 12.5 mg l^−1^. The concentrations of all of the elemental standards were obtained through fivefold serial dilution with 1% nitric acid.

## 3. Statistical analysis

The data were analysed by one-way analysis of variance (ANOVA). The significance level was set to *p* *<* 0.05. Hierarchical cluster analysis (HCA) was performed based on the Ward method to classify the eight different geographical origins of *A. elata*, and principal component analysis (PCA) was performed using SPSS v. 17.0.

## 4. Results and discussion

### 4.1. Basic chemical composition of *A. elata* buds from different geographical origins

The basic chemical composition such as ash, crude fibre, polysaccharides, total flavonoid and TS contents from the various sites are presented in table 1. The obtained results showed that the ash content ranged from 8.76% to 10.35%, and the sample 7 (S7) had the highest content (10.35%) and S8 had the lowest content (8.76%). The highest crude fibre content was found in S4, which was 11.07% and significantly higher than other samples (*p* *<* 0.05). Also, the lowest crude fibre contents were recorded in S3 and S6, which were 5.38% and 5.43%, respectively.

**Table 1.** Basic composition in *A. elata* buds of different geographical origins. TFC, total flavonoid content; TS, total saponins. Results are means ± s.d. of three replications. Different letters indicate significant differences (*p* < 0.05) in the samples from different geographical origins.

sample	ash (%)	crude fibre (%)	polysaccharides (mg g^−1^)	TFC (mg g^−1^)	TS (mg g^−1^)
S1	9.08 ± 0.16c	6.17 ± 0.16c	39.44 ± 0.28cd	4.81 ± 0.19e	20.01 ± 1.52bc
S2	9.62 ± 0.08b	6.50 ± 0.16c	33.85 ± 3.71d	4.06 ± 0.20e	17.94 ± 1.97cd
S3	9.90 ± 0.04b	5.38 ± 0.02d	37.18 ± 1.59cd	14.62 ± 0.56c	27.85 ± 2.31a
S4	9.81 ± 0.28b	11.07 ± 0.43a	44.17 ± 3.56bc	48.63 ± 0.26a	17.77 ± 0.34cd
S5	8.89 ± 0.19cd	6.55 ± 0.17c	52.48 ± 5.29a	24.19 ± 2.59b	13.96 ± 1.11e
S6	9.72 ± 0.07b	5.43 ± 0.13d	38.69 ± 6.50cd	13.38 ± 0.36cd	16.12 ± 2.00de
S7	10.35 ± 0.05a	6.15 ± 0.15c	46.79 ± 3.69ab	12.00 ± 0.29d	13.62 ± 0.90e
S8	8.76 ± 0.23d	8.22 ± 0.59b	37.72 ± 3.80cd	5.07 ± 0.19e	22.43 ± 1.39b

The highest polysaccharide content was in S5 (52.48 mg g^−1^), and the lowest content was in S2 (33.85 mg g^−1^). There was no significant difference in polysaccharide content among other samples (*p* *>* 0.05). The TFC was highest in S4 (48.63 mg g^−1^), but only 4.06 mg g^−1^ in S2. In general, plant polyphenols such as flavonoids and phenolics were biosynthesized through several pathways and formed a heterogeneous group [19]. Flavonoids, as critical secondary metabolites in the plant, exhibited various biological activities, such as anti-cancer and anti-oedema activities, free radical scavenging, prevention of coronary heart disease and inhibition of the arachidonic acid pathway to exert anti-inflammatory activity, etc. [20,21]. Our results indicated that S4 had the highest biological activities related to phenolic compounds.

The results also showed that the TS content in S3 was much higher than the other samples (27.85 mg g^−1^). The lowest contents were found in S5 and S7, which were 13.96 mg g^−1^ and 13.62 mg g^−1^, respectively. The total saponins of *A. elata* were approved as a plant extract drug by State Food and Drug Administration of China in 2002 (Approval number: B20020945), which was used to reduce enzymes, protect the liver and for anti-tumour effects [22]. *A. elata* leaves total saponins capsule (H drug, pharmaceutical name Z20131018) is a hospital preparation of the Second Affiliated Hospital of Heilongjiang University of Traditional Chinese Medicine; it is mainly used for the adjuvant treatment of cancer patients [23–25].

### 4.2. Phenolic composition of *A. elata* buds from different geographical origins

**Figure 1.**
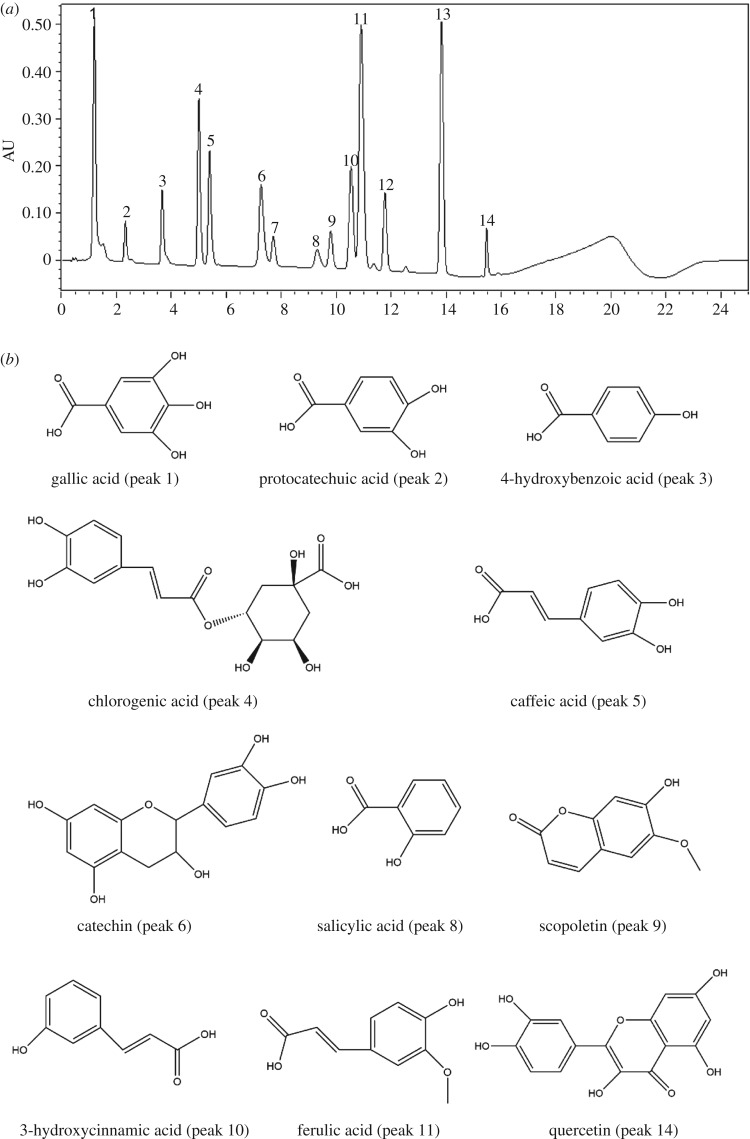
(*a*) UPLC chromatogram of 14 standard mixtures, peak 1: gallic acid; peak 2: protocatechuic acid; peak 3: 4-hydroxybenzoic acid; peak 4: chlorogenic acid; peak 5: caffeic acid; peak 6: catechin; peak 7: epicatechin; peak 8: salicylic acid; peak 9: scopoletin; peak 10: 3-hydroxycinnamic acid; peak 11: ferulic acid; peak 12: sinapic acid; peak 13: 4-hydroxycoumarin; peak 14: quercetin. (*b*) The structures of the phenolic compounds were detected.

The gradient method was optimized to provide separation of all the bioactive compounds by UPLC combined with LC-MS/MS. We measured 14 phenolic standards in this experiment, and 11 phenolic compounds have been identified (figure 1*a,b*).We clearly found that the compounds were unambiguously identified as gallic acid (peak 1), protocatechuic acid (peak 2), 4-hydroxybenzoic acid (peak 3), chlorogenic acid (peak 4), caffeic acid (peak 5), catechin (peak 6), epicatechin (peak 7), salicylic acid (peak 8), scopoletin (peak 9), 3-hydroxycinnamic acid (peak 10), ferulic acid (peak 11), sinapic acid (peak 12), 4-hydroxycoumarin (peak 13) and quercetin (peak 14).

The content of each analyte in different geographical origins of *A. elata* buds was calculated from the corresponding calibration curve and is summarized in table 2. Gallic acid and protocatechuic acid were estimated to be maximum in S1 and S2. S3 was rich in chlorogenic acid (0.96 mg g^−1^), quercetin (0.85 mg g^−1^) and protocatechuic acid (0.45 mg g^−1^). In addition, quercetin content was found to be maximum in S4 (11.71 mg g^−1^), which was 46.8-fold that of S2; chlorogenic acid (1.90 mg g^−1^) and salicylic acid (0.58 mg g^−1^) also had higher contents. Quercetin, gallic acid and chlorogenic acid contents were found as the major constituents in S5. With regard to S6 and S7, they were rich in quercetin and gallic acid contents. The maximum estimated quantities of quercetin, gallic acid and protocatechuic acid were, respectively, 3.4, 2.6 and 0.76 mg g^−1^ in S8.

**Table 2.** Phenolic compositions in *A. elata* buds of different geographical origins. Fer, Ferulic acid; Sal, Salicylic acid; Caf, Caffeic acid; Pro, Protocatechuic acid; 4-Hyd, 4-Hydroxybenzoic acid; Cat, Catechin; Sco, Scopoletin; Chl, Chlorogenic acid; Que, Quercetin; Gal, Gallic acid; 3-Hyd, 3-Hydroxycinnamic acid; TPC, total phenolic content. Results are means ± s.d. of three replications. Different letters indicate significant differences (*p* < 0.05) in the samples from different geographical origins. n.d., indicates not detected.

standard compound	quantity of the identified compounds (mg g^−1^)							
	S1	S2	S3	S4	S5	S6	S7	S8
Fer	0.01 ± 0.00b	0.01 ± 0.00b	0.02 ± 0.01b	0.06 ± 0.02a	0.01 ± 0.00b	n.d.	0.01 ± 0.01b	0.01 ± 0.00b
Sal	0.23 ± 0.08cd	0.12 ± 0.02e	0.22 ± 0.00cd	0.58 ± 0.04a	0.35 ± 0.04b	0.17 ± 0.02de	0.24 ± 0.01c	0.21 ± 0.04cd
Caf	0.16 ± 0.03cd	0.11 ± 0.01e	0.37 ± 0.01a	0.30 ± 0.04b	0.15 ± 0.05de	0.16 ± 0.03cd	0.17 ± 0.01cd	0.21 ± 0.01c
Pro	0.40 ± 0.02b	0.33 ± 0.08c	0.45 ± 0.02b	0.28 ± 0.02c	0.15 ± 0.02d	0.19 ± 0.02d	0.16 ± 0.00d	0.76 ± 0.02a
4-Hyd	0.10 ± 0.01ab	0.03 ± 0.00c	0.15 ± 0.09a	0.09 ± 0.01abc	0.11 ± 0.02ab	0.10 ± 0.01ab	0.10 ± 0.01ab	0.07 ± 0.01bc
Cat	0.04 ± 0.01c	0.02 ± 0.01c	0.18 ± 0.00a	0.16 ± 0.02a	0.11 ± 0.02b	0.03 ± 0.01c	0.04 ± 0.01c	0.05 ± 0.00c
Sco	0.13 ± 0.05c	0.07 ± 0.02d	n.d.	0.36 ± 0.04a	n.d.	0.07 ± 0.01d	0.14 ± 0.01c	0.20 ± 0.01b
Chl	0.23 ± 0.01de	0.11 ± 0.02e	0.96 ± 0.15b	1.90 ± 0.12a	1.15 ± 0.24b	0.34 ± 0.05cd	0.45 ± 0.01c	0.17 ± 0.08de
Que	0.32 ± 0.02c	0.25 ± 0.09c	0.85 ± 0.17c	11.71 ± 1.58a	4.03 ± 0.10b	0.62 ± 0.18c	0.79 ± 0.00c	3.40 ± 0.18b
Gal	1.99 ± 0.09a	1.69 ± 0.12b	n.d.	n.d.	1.38 ± 0.07c	1.80 ± 0.08b	0.73 ± 0.03d	2.06 ± 0.12a
3-Hyd	0.05 ± 0.01b	0.03 ± 0.00b	0.05 ± 0.03b	0.13 ± 0.04a	0.04 ± 0.01b	0.05 ± 0.01b	0.04 ± 0.02b	0.05 ± 0.00b
TPC	4.91 ± 0.12bc	3.67 ± 0.19de	5.54 ± 0.26b	14.55 ± 1.27a	3.84 ± 1.10cde	2.86 ± 0.32e	4.49 ± 0.06bcd	4.95 ± 0.12bc

The comprehensive quantitative analysis results indicated that quercetin was detected as the major constituent, especially in S4 (11.7 mg g^−1^). Gallic acid was higher in S1, S2, S5, S6, S7 and S8. However, it was not detected in S3 and S4. In addition, the protocatechuic acid content was much higher in S1, S2, S3, S4 and S8. The highest total amount of phenolic compounds was found in S4, which was 14.55 mg g^−1^ and much higher than other samples (*p* *<* 0.05). The lowest content was 2.86 mg g^−1^ in S6. These compounds showed different contents supposedly because of the location where the samples were collected as well as the dominant climatic and environmental factors [26]. From table 2, gallic acid, protocatechuic acid, quercetin and chlorogenic acid were found to be high in the eight different geographical origins of *A. elata*. Therefore, it is speculated that this may be related to the anti-tumour, antioxidant and anti-inflammatory effects of *A. elata*, which need to be further studied.

### 4.3. Hierarchical cluster and principal component analysis of phenolic compounds

**Figure 2.**
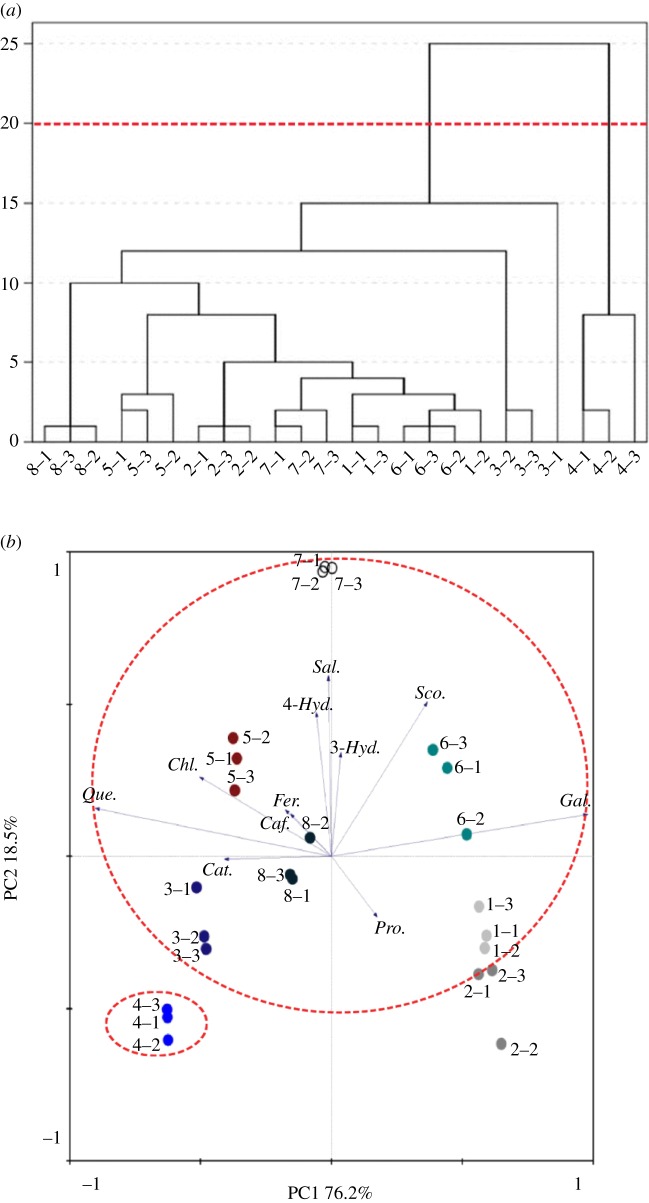
(*a*) Hierarchical cluster analysis of phenolic compounds in *A. elata* buds of different geographical origins, (*b*) principal component analysis of phenolic compounds in *A. elata* buds of different geographical origins Fer, Ferulic acid; Sal, Salicylic acid; Caf, Caffeic acid; Pro, Protocatechuic acid; 4-Hyd, 4-Hydroxybenzoic acid; Cat, Catechin; Sco, Scopoletin; Chl, Chlorogenic acid; Que, Quercetin; Gal, Gallic acid; 3-Hyd, 3-Hydroxycinnamic acid.

To evaluate the possible similarities and relationship of the phenolic compounds in *A. elata* buds among the eight different geographical origins, HCA was performed based on the phenolic compounds. The HCA results were presented in the form of a dendrogram in figure 2*a*. Based on this analysis result, eight different geographical origins of *A. elata* buds were classified into two groups. The first group consisted of seven samples (S1, S2, S3, S5, S6, S7 and S8). The second group only included S4, which is mainly because of its exceptionally high level of quercetin (11.7 mg g^−1^) and TPC (14.55 mg g^−1^).

In addition, PCA classification confirmed the results of cluster analysis (figure 2*b*). The PCA result showed that the first component explained 76.2% of the total variation, and the second principal component explained 18.5% of the total variation. It clearly showed that S1 and S2 were rich in protocatechuic acid, S6 was rich in gallic acid, and S4 formed a single group characterized by higher quantities of quercetin.

### 4.4. Mineral elements in *A. elata* buds from different geographical origins

The mineral content in *A. elata* buds of the eight different geographical origins of *A. elata* buds is presented in table 3. The obtained results showed that the Ca content ranged from 801.47 to 3496.73 (μg g^−1^), Mg content ranged from 2577.53 to 3593.53 (μg g^−1^), Fe content ranged from 39.58 to 115.01 (μg g^−1^), Sr content ranged from 2.44 to 12.57 (μg g^−1^), Co content ranged from 0 to 0.34 (μg g^−1^), Mn content ranged from 25.66 to 214.32 (μg g^−1^), Ni content ranged from 1.25 to 6.43 (μg g^−1^), Cu content ranged from 20.1 to 46.73 (μg g^−1^) and Zn content ranged from 44.05 to 73.51 (μg g^−1^).

**Table 3.** Mineral elements in *A. elata* buds of different geographical origins. Results (μg g^−1^) are means ± s.d. of three replications. Different letters indicate significant differences (*p* < 0.05) in the samples from different geographical origins. n.d., indicates not detected.

sample	Co	Ni	Cu	Zn	Sr
S1	0.04 ± 0.00b	4.77 ± 0.16ab	26.66 ± 1.06d	57.57 ± 2.45c	9.56 ± 0.20b
S2	0.02 ± 0.00b	2.38 ± 0.05ab	41.79 ± 2.27b	63.01 ± 0.62b	7.70 ± 0.31c
S3	n.d.	2.35 ± 0.24ab	26.99 ± 3.50d	44.05 ± 3.50e	2.44 ± 0.83e
S4	0.34 ± 0.19a	5.95 ± 0.23a	31.69 ± 3.01c	50.07 ± 3.09d	5.52 ± 1.05d
S5	0.05 ± 0.00b	3.65 ± 0.21ab	46.73 ± 1.32a	65.28 ± 2.71b	10.25 ± 0.43b
S6	n.d.	3.69 ± 0.83ab	39.58 ± 1.45b	65.99 ± 1.82b	7.91 ± 0.52c
S7	0.04 ± 0.04b	6.43 ± 6.04a	40.63 ± 1.06b	73.51 ± 4.91a	5.75 ± 0.53d
S8	n.d.	1.25 ± 0.14b	20.10 ± 1.53e	46.88 ± 3.01de	12.57 ± 0.99a

sample	Ca	Mg	Mn	Fe
S1	2981.40 ± 204.26b	3127.53 ± 204.21bc	158.39 ± 9.08b	98.95 ± 3.48b
S2	2662.07 ± 101.00c	3008.20 ± 35.16cd	86.07 ± 1.56c	112.37 ± 7.33a
S3	801.47 ± 90.55f	2518.20 ± 259.42f	25.66 ± 1.33f	47.91 ± 2.61c
S4	2331.40 ± 77.87d	3593.53 ± 114.01a	214.32 ± 19.79a	55.72 ± 7.74c
S5	2620.07 ± 181.02c	2949.53 ± 128.08cd	144.38 ± 7.27b	115.01 ± 6.18a
S6	1573.93 ± 115.91e	2577.53 ± 86.19ef	44.52 ± 0.62e	39.58 ± 2.48b
S7	1396.87 ± 40.06e	2832.60 ± 121.10de	53.95 ± 3.61de	105.89 ± 12.35ab
S8	3496.73 ± 145.14a	3352.20 ± 135.51ab	59.71 ± 2.93d	95.75 ± 2.48b

The contents of Fe and Cu in the S5 were higher than others. The Mg, Mn, Co and Ni contents were estimated to be maximum in S4, and S7 was rich in Zn content. In addition, the maximum estimated quantities of Ca and Sr were found in S8. However, Co was not detected in S3, S7 and S8. These minerals are necessary for the regulation of various functions in the human body. For example, Ca and Mg are important for bone health, Fe is the most vital component of haemoglobin, and Mn and Zn are responsible for regulating the activities of many enzymes [27]. In this research, we found that the buds of *A. elata* had high levels of some important minerals such as Ca, Mg and Zn. Thus, the buds of *A. elata* have important nutritional benefits for humans.

In summary, the present study compared the total flavonoids, total saponins, phenolic compounds and mineral elements contents in the buds of *A. elata* collected from eight different geographical regions in China, and found that the chemical composition in the buds of *A. elata* was obviously affected by the geographical origin. The results could provide an important theoretical basis of quality evaluation of *A. elata* buds in the food production field.

## Permission to carry out fieldwork

Permission for collection of *A. elata* buds was granted to Northeast Forestry University. These eight sampling sites have cooperation with the university. We did our experiments in the university.

## Data accessibility

Our data are deposited at Dryad Digital Repository: http://dx.doi.org/10.5061/dryad.m3jf509 [28].

## References

[1] Wang M, Xu X, Xu H, Wen F, Zhang X, Sun H, Yao F, Sun G, Sun X 2014 Effect of the total saponins of *Aralia elata* (Miq) Seem on cardiac contractile function and intracellular calcium cycling regulation. J. Ethnopharmacol. 155, 240–247. (10.1016/j.jep.2014.05.024) 24875646

[2] Zhou CC, Shi HB, Li WT 1984 Anti-inflammatory action of *Aralia continentalis* Kitagwa extracts. Chinese Herbal Medicine 15, 21–25.

[3] Chung YS, Choi YH, Lee SJ, Choi SA, Lee JH, Kim H, Hong EK 2005 Water extract of *Aralia elata* prevents cataractogenesis in vitro and in vivo. J. Ethnopharmacol. 101, 49–54. (10.1016/j.jep.2005.03.020) 15905053

[4] Du S, Chi B 2005 Protective effects of saponins derived from *Aralia Elata* (Miq) Seem on alcoholic liver disease in rats. J. Jilin University 31, 64–67.

[5] Zhang J, Wang H, Xue Y, Zheng Q 2013 Cardioprotective and antioxidant activities of a polysaccharide from the root bark of *Aralia elata* (Miq.) Seem. Carbohydr. Polym. 93, 442–448. (10.1016/j.carbpol.2012.12.0480) 23499081

[6] Jiao Z, Deng JC, Li GK, Zhang ZM, Cai ZW 2010 Study on the compositional differences between transgenic and non-transgenic papaya (*Carica papaya* L.). J. Food Compos. Anal. 23, 640–647. (10.1016/j.jfca.2010.03.004)

[7] Saeed F, Arshad MU, Pasha I, Naz R, Batool R, Khan AA, Nasir MA, Shafique B 2014 Nutritional and phyto-therapeutic potential of papaya (*Carica papaya* Linn.): an overview. Int. J. Food Prop. 17, 1637–1653. (10.1080/10942912.2012.709210)

[8] Sumczynski D, Kotaskova E, Orsavova J, Valasek P 2017 Contribution of individual phenolics to antioxidant activity and in vitro digestibility of wild rices (*Zizaniaaquatica L.*). Food Chem. 218, 107–115. (10.1016/j.foodchem.2016.09.060) 27719885

[9] Milosevic-Dordevic O, Grujicic D, Radovic M, Vukovic N, Zizic J, Markovic S 2015 In vitro chemoprotective and anticancer activities of propolis in human lymphocytes and breast cancer cells. Arch. Biol. Sci. 67, 571–581. (10.2298/ABS141013019M)

[10] Dordas C 2008 Role of nutrients in controlling plant diseases in sustainable agriculture: a review. Agron. Sustainable Dev. 28, 33–46. (10.1051/agro:2007051)

[11] Charpentier M, Oldroyd GE 2013 Nuclear calcium signaling in plants. Plant Physiol. 163, 496 (10.1104/pp.113.220863) 23749852 PMC3793031

[12] Sieprawska A, Filek M, Walas S, Tobiasz A, Mrowiec H, Miszalski Z 2014 Does micro- and macroelement content differentiate grains of sensitive and tolerant wheat varieties? Acta Physiol. Plant. 36, 3095–3100. (10.1007/s11738-014-1666-x)

[13] Song SJ, Nakamura N, Ma CM, Hattori M, Xu SX 2001 Five saponins from the root bark of *Aralia elata*. Phytochemistry 56, 491–497. (10.1016/S0031-9422(00)00379-4) 11261582

[14] Tomatsu M, Ohnishi-Kameyama M, Shibamoto N 2003 A new cytotoxic protein from *Aralia elata*, inducing apoptosis in human cancer cells. Cancer Lett. 199, 19–25. (10.1016/S0304-3835(03)00348-3) 12963119

[15] Zhang M, Liu GS, Tang S, Song S, Yamashita K, Manabe M 2006 Effect of five triterpenoid compounds from the buds of *Aralia elata* on stimulus-induced superoxide generation, tyrosyl phosphorylation and translocation of cytosolic compounds to the cell membrane in human neutrophils. Planta Med. 72, 1216–1222. (10.1055/s-2006-951679) 17021995

[16] Dubois M, Gilles KA, Hamilton JK, Rebers PA, Smith F 1956 Colorimetric method for determination of sugars and related substances. Anal. Chem. 28, 350–356. (10.1021/ac60111a017)

[17] Zheng YF, Zhang Q, Liu XM, Ma L, Lai F 2016 Extraction of polysaccharides and its antitumor activity on *Magnolia kwangsiensis* Figlar & Noot. Carbohydr. Polym. 142, 98–104. (10.1016/j.carbpol.2016.01.039) 26917379

[18] Chandrasekara A, Shahidi F 2010 Content of insoluble bound phenolics in millets and their contribution to antioxidant capacity. J. Agri. Food Chem. 58, 6706–6714. (10.1021/jf100868b) 20465288

[19] Gharibi S, Tabatabaei BES, Saeidi G 2015 Comparison of essential oil composition, flavonoid content and antioxidant activity in eight *Achillea* species. J. Essent. Oil Bear. Plants 18, 1382–1394. (10.1080/0972060X.2014.981600)

[20] Mojzisová G, Kuchta M 1994 Dietary flavonoids and risk of coronary heart disease. Nutr. Rev. 52, 59–61. 8183470

[21] Middleton E, Kandaswami C, Theoharides TC 2000 The effects of plant flavonoids on mammalian cells: implications for inflammation, heart disease, cancer. Pharmacol. Rev. 52, 673–751. 11121513

[22] Wang Z, Wu Q, Meng Y, Sun Y, Wang Q, Yang C, Wang Q, Yang B, Kuang H 2015 Determination and pharmacokinetic study of two triterpenoid saponins in rat plasma after oral administration of the extract of *Aralia elata* leaves by UHPLC-ESI-MS/MS. J. Chromatogr. B-Analyt. Technol. Biomed. Life Sci. 985, 164–171. (10.1016/j.jchromb.2015.01.036) 25687802

[23] Kuang HX, Wang ZB, Wang QH, Yang BY, Xiao HB, Okada Y, Okuyama T 2013 Triterpene glucosides from the leaves of *Aralia elata* and their cytotoxic activities. Chem. Biodivers. 10, 703–710. (10.1002/cbdv.201200087) 23576356

[24] Kuang HX, Sun H, Zhang N, Okada Y, Okuyama T 1996 Two new saponins, congmuyenosides A and B, from the leaves of *Aralia elata* collected in Heilongjiang, China. Chem. Pharm. Bull. 44, 2183–2185. (10.1248/cpb.44.2183) 8945786

[25] Sun Y, Li B, Lin X, Xue J, Wang Z, Zhang H, Jiang H, Wang Q, Kuang H 2017 Simultaneous determination of four triterpenoid saponins in *Aralia elata* leaves by HPLC-ELSD combined with hierarchical clustering analysis. Phytochem. Anal. 28, 202–209. (10.1002/pca.2662) 28071864

[26] Rahimmalek M, Bahreininejad B, Khorrami M, Tabatabaei BES 2009 Genetic variability and geographic differentiation in *Thymus daenensis* subsp. *daenensis*, an endangered medicinal plant, as revealed by inter simple sequence repeat (ISSR) markers. Biochem. Genet. 47, 831–842. (10.1007/s10528-009-9281-z) 19657729

[27] Taskaya L, Chen YC, Beamer S, Tou JC, Jaczynski J 2009 Compositional characteristics of materials recovered from whole gutted silver carp (*Hypophthalmichthys molitrix*) using isoelectric solubilization/precipitation. J. Agri. Food Chem. 57, 4259–4266. (10.1021/jf803974q) 19368395

[28] Qi M, Peng X, Hua X, Yan X, Lin J 2018 Data from: Comparison of chemical composition in the buds of *Aralia elata* from different geographical origins of China *Dryad Digital Repository*. (10.5061/dryad.m3jf509) PMC6124108 30225063

